# A dialectical behavior therapy skills training smartphone app for recurrent binge eating: a randomized clinical trial

**DOI:** 10.1017/S0033291724002800

**Published:** 2024-12

**Authors:** Jake Linardon, Cleo Anderson, Zoe McClure, Claudia Liu, Mariel Messer, Hannah K. Jarman, Matthew Fuller-Tyszkiewicz

**Affiliations:** SEED Lifespan Strategic Research Centre, School of Psychology, Faculty of Health, Deakin University, Geelong, Australia

**Keywords:** dialectical behavior therapy, digital health, eating disorders, smartphones

## Abstract

**Background:**

Dialectical behavior therapy (DBT) is a specialized treatment that has a growing evidence base for binge-spectrum eating disorders. However, cost and workforce capacity limit wide-scale uptake of DBT since it involves over 20 in-person sessions with a trained professional (and six sessions for guided self-help format). Interventions translated for delivery through modern technology offer a solution to increase the accessibility of evidence-based treatments. We developed the first DBT-specific skills training smartphone application (*Resilience*: *eDBT*) for binge-spectrum eating disorders and evaluated its efficacy in a randomized clinical trial.

**Method:**

Participants reporting recurrent binge eating were randomized to *Resilience* (*n* = 287) or a waitlist (*n* = 289). Primary outcomes were objective binge eating episodes and global levels of eating disorder psychopathology. Secondary outcomes were behavioral and cognitive symptoms, psychological distress, and the hypothesized processes of change (mindfulness, emotion regulation, and distress tolerance).

**Results:**

Intention-to-treat analyses showed that the intervention group reported greater reductions in objective binge eating episodes (incidence rate ratio = 0.69) and eating disorder psychopathology (*d* = −0.68) than the waitlist at 6 weeks. Significant group differences favoring the intervention group were also observed on secondary outcomes, except for subjective binge eating, psychological distress, and distress tolerance. Primary symptoms showed further improvements from 6 to 12 weeks. However, dropout rate was high (48%) among the intervention group, and engagement decreased over the study period.

**Conclusion:**

A novel, low-intensity DBT skills training app can effectively reduce symptoms of eating disorders. Scalable apps like these may increase the accessibility of evidence-based treatments.

## Introduction

Difficulties with regulating emotional states are implicated in the development and persistence of binge eating. According to affect regulation models (Heatherton & Baumeister, 1991; Stice, Nemeroff, & Shaw, 1996), binge eating is a maladaptive coping strategy used to escape, avoid, or manage negative affect. Over time, this maladaptive behavior becomes a conditioned response that is maintained through negative reinforcement. Since there is robust evidence from both experimental (Russell, Haynos, Crow, & Fruzzetti, 2017) and longitudinal (McClure, Messer, Anderson, Liu, & Linardon, 2022) designs linking emotion dysregulation with the onset and maintenance of binge eating, a key aim of many established early intervention and treatment protocols for eating disorders is to foster healthy coping strategies (Fairburn, 2008).

One approach that places considerable emphasis on emotion dysregulation is dialectical behavior therapy (DBT; Linehan, 1993). DBT was initially developed for borderline personality disorder, where the original protocol was based on a multimodal approach comprised of weekly individual psychotherapy, group skills training, 24-h telephone consultation, and a therapist consultation team (Linehan et al., 2006). DBT has since been adapted for use to treat binge-eating disorder and bulimia nervosa (Chen, Yiu, & Safer, 2017). In the traditional therapist-led format, DBT includes 20 outpatient sessions of either individual or group treatment that covers three core skill areas: mindfulness, emotion regulation, and distress tolerance. This therapist-led version of DBT has since been translated to lower intensity formats, including six sessions of guided self-help sessions delivered in face-to-face (Carter, Kenny, Singleton, Van Wijk, & Heath, 2020) or telephone format (Masson, von Ranson, Wallace, & Safer, 2013), where the central role of the facilitator is to check progress, clarify concepts, and encourage utilization of the three key skill domains.

Growing evidence supports the clinical effectiveness of DBT for eating disorders (Linardon, Fairburn, Fitzsimmons-Craft, Wilfley, & Brennan, 2017). Randomized clinical trials (RCTs) have shown standard DBT to be more effective than both passive (waitlist) and active comparisons (supportive therapy) for symptom reduction and abstinence among individuals with bulimia nervosa (Safer, Telch, & Agras, 2001) or binge-eating disorder (Safer, Robinson, & Jo, 2010). There is also evidence that 6 and 12 month outcomes do not differ among patients with binge-spectrum eating disorders who were treated with DBT relative to standard cognitive-behavioral therapy (CBT) – the leading evidence-based treatment for eating disorders (Chen et al., 2016).

Despite the availability of empirically validated treatments like DBT, few people in need have access to them (Weissman & Rosselli, 2017). The reasons for this center around high costs of treatment, insufficient number of professionals trained in these specialized approaches, geographical isolation from established treatment services, perceived stigma associated with help-seeking, and privacy concerns (Ali et al., 2017). Efficient and cost-effective methods to deliver interventions at scale are required to address the treatment gap and reduce the burden of disease associated with eating disorders.

Smartphone technology offers a viable solution to broaden the dissemination of evidence-based treatments. Smartphones are among the most rapidly adopted technological innovation in modern times, with nearly 7 billion people owning a smartphone and keeping it within arm's reach at almost all times (Poushter, 2016). Components of evidence-based treatments can be translated for delivery via downloadable applications (apps), which can be accessed anytime, anywhere, and in critical moments without the need for ongoing professional support (Linardon et al., 2024; Torous et al., 2019). In-built monitoring mechanisms that capture vast amounts of data can be rapidly analyzed to deploy flexible and personalized intervention resources in real time (Torous et al., 2021), something that is not possible in conventional treatment.

The available research investigating the viability and clinical utility of app-based interventions for eating disorders is encouraging. Prior survey studies show that 9 in 10 people with an eating disorder report willingness to use an app for symptom management (Anderson, Fuller-Tyszkiewicz, Messer, & Linardon, 2024b) with one in four preferring it over face-to-face treatment (Linardon, Messer, Lee, & Rosato, 2020a). This may reflect the fact that people with eating disorders typically express ambivalence to change (Ålgars et al., 2015), with apps enabling the person to take control over their treatment and approach it at their own pace. RCTs show that apps based on traditional CBT principles delivered as a stand-alone intervention (Linardon, Shatte, Rosato, & Fuller-Tyszkiewicz, 2022) or an adjunct to more intensive services (Hildebrandt et al., 2020) are efficacious in samples with binge-eating disorder. Further research designed to investigate the utility of app-based interventions for eating disorders – particularly those that diverge from second-wave CBT principles – is needed to build on this evolving evidence base.

We recently developed the first DBT skills training app (*Resilience: eDBT*) for eating disorders and gathered preliminary evidence on its usability, acceptability, and perceived helpfulness (Anderson, Fuller-Tyszkiewicz, Messer, & Linardon, 2024a). Specifically, *Resilience* received a mean Systems Usability Scale score of 85.5 from 10 end-users, which far exceeded the cut-off of 68 that is indicative of acceptable usability. Participants also noted a number of key strengths of *Resilience*, including its visual design, intuitive instructions, and engaging content. The present research reports the results of a RCT testing the efficacy of *Resilience* in individuals with recurrent binge eating. It was hypothesized that participants allocated to *Resilience* would experience greater improvements in symptoms and the purported change mechanisms than participants allocated to the control group.

## Method

### Design

A two-armed, fully remote RCT was conducted comparing the *Resilience* app against a waitlist control condition. Assessments were conducted at baseline, 6 weeks, and 12 weeks from baseline. This study received ethical clearance from Deakin University and all participants provided informed consent. The trial was pre-registered in the Australian New Zealand Clinical Trials registry (ACTRN12624000158561). There were no deviations to the pre-registered protocol. We adhered to the CONSORT guidelines for RCTs (see online Supplementary Table S1).

### Population and recruitment

We recruited participants in February 2024 through advertisements distributed on the authors' online educational platform for eating disorders. This platform is composed of an open-access website and corresponding social media accounts that displays passive information about eating disorders. The platform has attracted close to 1 million users globally since its inception in 2019, with 8 in 10 reporting their main reason for visiting the platform was to obtain help. A previous survey on a sample of platform visitors showed that one in two met criteria for a clinically significant eating disorder (Linardon, Rosato, & Messer, 2020b).

Those who responded to trial advertisements completed a brief screening questionnaire to determine their eligibility. Participants were eligible if they (i) were aged 18 years or over, (ii) had access to a smartphone, and (iii) reported the presence of recurrent binge eating, which we defined as engaging in at least one objective binge eating episode a fortnight (2 weeks), on average, over the last 3 months, consistent with recent trials (Linardon, Shatte, McClure, & Fuller-Tyszkiewicz, 2023; Messer, Fuller-Tyszkiewicz, Liu, Anderson, & Linardon, 2024). Those who met eligibility criteria went on to complete the baseline assessment battery.

### Randomization

Upon completing the baseline assessment, participants were randomized at a ratio of 1:1 and a block size of 2 using an automated computer-based random number sequence generated through Qualtrics. Since the randomization process was completely automated, upcoming allocations were concealed from the research team and participants.

### Conditions

#### Intervention

The *Resilience eDBT* app was delivered through either iOS or Android devices. *Resilience* was built through a user-centered framework, where samples drawn from the target population were involved in its conception and offered rigorous feedback on its design, features, and usability at key stages of the developmental process (see Anderson et al., 2024a, 2024b).

The app is based on empirically supported DBT skills training protocols (Chen et al., 2017; Safer, Adler, & Masson, 2018) that targets binge eating through use of healthy coping strategies. The app is structured in three main sections: modules, skills, and reflections. The five modules are educative in nature; they teach users critical concepts, provide a rationale for the core DBT skills, illustrate skill use examples through case vignettes, and offer ‘walk-through’ example skills for users to understand. The five modules include: (1) Preparing for Change; (2) Connecting the Dots; (3) Mindfulness; (4) Emotion Regulation; and (5) Distress Tolerance. Users are required to complete the first two modules before the remaining modules unlock, as modules 1 and 2 provide the theoretical foundations for DBT skills training and introduces users to two fundamental DBT techniques (Diary Card and Chain Analysis) that need to be learnt before progressing to the three skill areas. Users were encouraged to complete the modules at a self-suited pace for the duration of the trial, and each module took between 30 and 90 min to fully complete.

The skills component offered a menu of brief activities that encompass either mindfulness, emotion regulation, or distress tolerance skills. These skills are designed to encourage users to take an active role in critical moments to de-escalate urges or prevent binge-eating behaviors from occurring. Each skill requires a very brief interactions with the app (1–5 min), because it is recognized that people tend to use health-related apps in short bursts, especially in high-risk situations (e.g. in moments of stress; Mohr et al., 2017).

**Figure 1.**
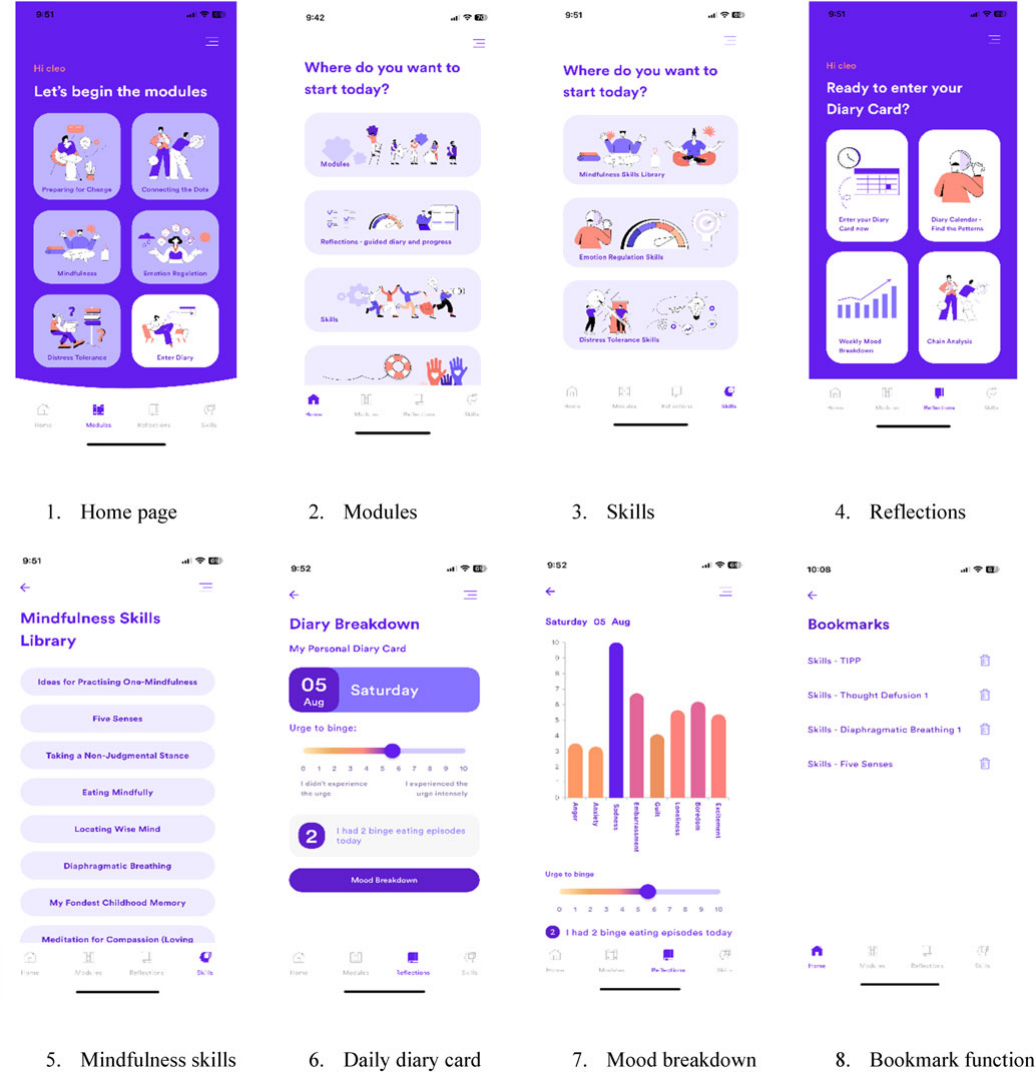
Screenshots of the *Resilience: eDBT* app.

The reflections component offers a daily digital diary card. The diary card enables assessment of thoughts, feelings, and behaviors to help users understand the connection between how different internal states can precipitate binge eating. Users can log their intensity of eight different emotions (e.g. excitement, anger, anxiety), their urge to binge eat, and the number of daily binge episodes. These inputted data are graphically presented on a daily, weekly, and fortnightly (2 weeks) basis to help users identify key patterns, track their progress, and understand what skills to prioritize to facilitate change. See Fig. 1 for screenshot examples of the *Resilience* app.

Other functionalities were included to enhance the user experience. Following gamification principles (Brown et al., 2016), after completing certain in-app tasks users received virtual badge achievements as incentive for continued engagement. Similarly, users were provided with daily opt-in notification prompts to complete the diary cards and engage with the app. A bookmark function was also added, enabling users to store activities, skills, or concepts in one place for ease of access. A log of past use was also included, allowing users to track their prior activity and continue where they left off.

Content was delivered in multimedia formats, including written text, images, videos, and audio recordings. The app was delivered in a self-guided format. In addition to in-app reminders, participants were also sent weekly automated emails encouraging continued app use. Participants allocated to immediate access could engage with the app for the entire duration of the trial.

#### Waitlist control group

Participants allocated to the waitlist control group were told that they would receive access to *Resilience* at 6 weeks. They were instructed to view the educational material presented on the authors' website https://breakbingeeating.com/ in the meantime.

### Assessments

#### Background characteristics

Participants at baseline were asked to indicate their age, gender, race, employment status, current and past diagnosis of an eating, anxiety, depressive, substance abuse, and personality disorder, and whether there were currently receiving any treatment for eating or body image issues.

#### Primary outcomes

There were two pre-registered primary outcomes. The first was the frequency of objective binge eating episodes experienced over the past 28 days, assessed by an individual item (‘How many times over the past 28 days did eat a large amount of food in a short-period and at the same time felt a sense of loss of control’). The second was the global score derived from the Eating Disorder Examination Questionnaire (EDE-Q; Fairburn & Beglin, 1994). The global score represents a measure of eating disorder psychopathology. It is calculated by averaging the eating concern, weight concern, shape concern, and dietary restraint subscale of the EDE-Q. Items are rated along a 7-point scale, and responses are averaged to produce a scale score. McDonald's omega (*ω*) was >0.85 across all time points.

#### Secondary outcomes

Secondary outcomes included the frequency of subjective binge eating and compensatory behaviors (laxative, self-induced vomiting, and driven exercise) episodes experienced over the past 28 days, as well as the shape concern (*ω* > 0.81), weight concern (*ω* > 0.83), eating concern (*ω* > 0.88), and dietary restraint (*ω* > 0.80) subscales of the EDE-Q. General psychological distress was also assessed via the total score (*ω* > 0.91) from 4-item Patient Health Questionnaire (Kroenke, Spitzer, Williams, & Löwe, 2009). We also assessed DBT mechanisms as secondary outcomes, including mindfulness (*ω* > 0.92) using the 15-item Five Factor Mindfulness Questionnaire (Baer et al., 2008), emotion regulation (*ω* > 0.87) using the 16-item Difficulties in Emotion Regulation Scale (Bjureberg et al., 2016), and distress tolerance (*ω* > 0.94) using the 4-item Distress Tolerance Scale (Garner et al., 2018).

#### Negative effects

Two items were used to assess negative effects. One item asked participants to indicate whether working with *Resilience* led to an aggravation of symptoms they had before, and the other asked participants to indicate whether working with *Resilience* led to new psychological complaints never experienced before. Response options were either ‘yes’ or ‘no’, and those who responded with ‘yes’ could elaborate through a free text response. Participants were also provided with the contact details of the research team (which included psychologists) in the unanticipated event that negative effects arose during the trial period. Participants were free to contact our team at any moment to report or discuss any adverse events. No such instances occurred.

### Sample size calculation

The required sample size was powered with the following assumptions: (1) a medium post-test between-group difference (*d* = 0.50) based on an anticipated yet conservative effect according to recent trials of app-based interventions for recurrent binge eating (Linardon et al., 2023); (2) power set to 0.80; (3) alpha set to 0.05 (two-tailed); (4) expected attrition rate of 40%; and (5) an allocation ratio of 1:1. Under these assumptions, we required a minimum of 107 participants per group, which was far exceeded in the present trial.

### Statistical analyses

Analyses were conducted with Stata version 18. Following intention-to-treat (ITT) principles, participant data were analyzed according to allocated groups at baseline. Mixed models were used to evaluate efficacy for primary and secondary outcomes, with time coded for comparisons with baseline as reference for immediate post-test assessments (baseline = 0, post-intervention = 1), group coded with waitlist as reference (waitlist control = 0, intervention = 1), and the group × time interaction terms as tests of efficacy of intervention relative to the waitlist control group at post-test. For tests of stability of change (6 to 12 weeks for intervention group) and intervention effect (6 to 12 weeks for the waitlist control group) models were run separately by group. A Gaussian distribution was assumed for all outcomes, except for frequencies of objective binge eating, subjective binge eating, and compensatory behaviors, for which a Poisson distribution was used instead.

In these models (at both 6 and 12 weeks), missing data were handled using multiple imputations with 50 imputations. However, as this approach makes an untestable assumption that missingness is ignorable, sensitivity analyses were conducted to evaluate the robustness of the observed results to the possible presence of non-ignorable patterns of missingness (not missing at random; NMAR). Pattern mixture models via the mimix package (Cro, Morris, Kenward, & Carpenter, 2016) were used to conduct the sensitivity analyses on the primary outcome variables. Several plausible NMAR patterns were tested with mimix: (1) last mean carried forward; (2) jump to reference; and (3) copy increments in reference (see Linardon et al., 2022). Fifty imputations were also undertaken per model.

Standardized mean differences were calculated per Feingold (2017) for continuous outcomes, and incidence rate ratios (IRRs) were used for count outcome. For standardized mean differences, values of 0.20, 0.50, and 0.80 represent small, moderate, and large effects, respectively (Cohen, 1992).

## Results

### Participant characteristics

**Figure 2.**
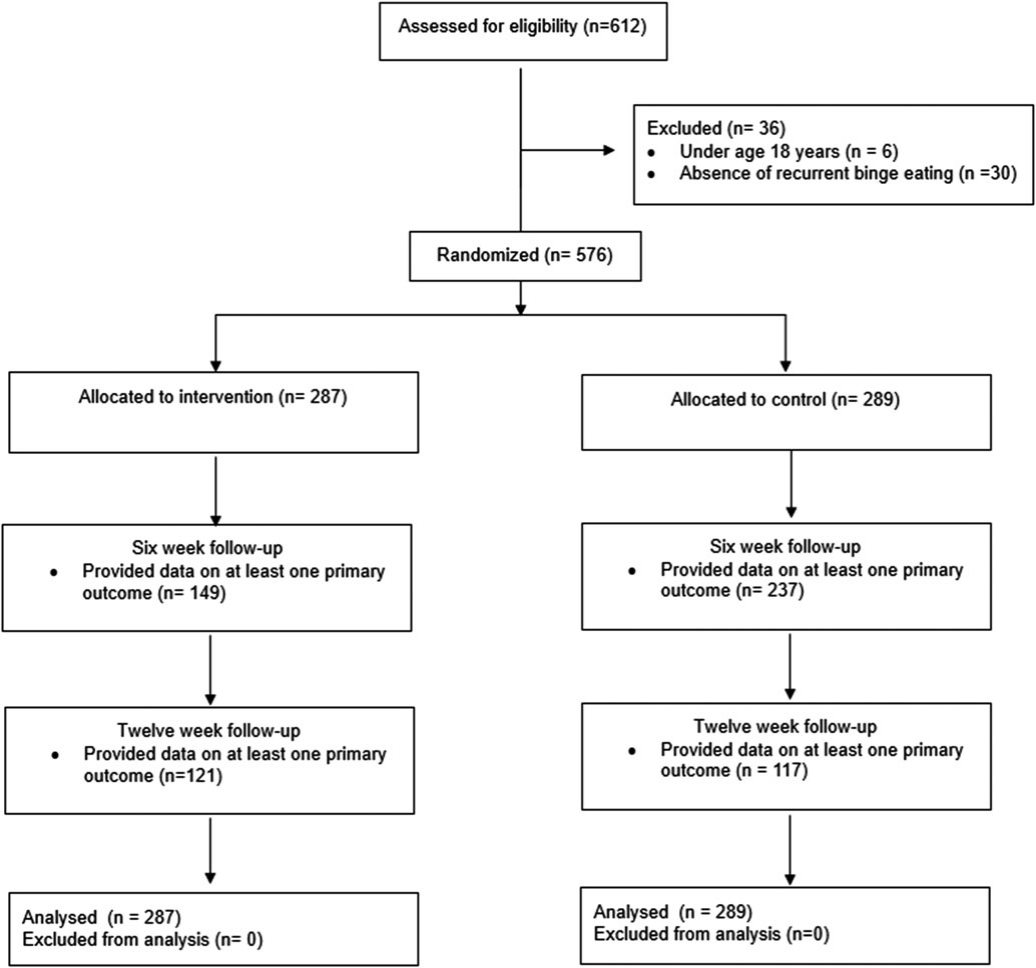
Flow of participants throughout the study.

A total of 576 participants were randomized to the intervention (*n* = 287) or waitlist control (*n* = 289) group (Fig. 2). For the total sample, the mean EDE-Q global score at baseline was 3.72 (s.d. = 1.05), which is within one standard deviation of clinical norms (Aardoom, Dingemans, Op't Landt, & Van Furth, 2012). The mean number of objective binge eating episodes over the past month at baseline was 14.89 (s.d. = 11.63), with 96% of the sample reporting at least one episode per week on average, which is consistent with diagnostic threshold used to define binge-eating disorder.

Table 1 presents the baseline characteristics of participants. The two study conditions did not significantly differ on any baseline variable.

**Table 1.** Baseline characteristics of participants

Variable	Intervention (*n* = 287)	Waitlist (*n* = 289)	Test statistic	Effect size
Age	39.75 (12.48)	38.56 (11.95)	1.17	0.09
Gender			0.74	0.03
Women	268 (93.4%)	265 (91.7%)		
Man	18 (6.3%)	22 (7.6%)		
Gender diverse	1 (0.3%)	2 (0.7%)		
Race and ethnicity			7.07	0.11
White	257 (92.4%)	245 (86.3%)		
Black	2 (0.7%)	4 (1.4%)		
Hispanic	5 (1.8%)	10 (3.5%)		
Asian	11 (4.0%)	16 (5.6%)		
Pacific Islander	0 (0%)	3 (1.1%)		
Mixed	3 (1.0%)	6 (2.1%)		
Other	10 (3.5%)	9 (3.1%)		
Employment			5.69	0.09
Unemployed	12 (4.2%)	8 (2.8%)		
Student	17 (5.9%)	25 (8.7%)		
Part-time	42 (14.6%)	45 (15.6%)		
Full-time	173 (60.3%)	182 (63.0%)		
Retired	19 (6.6%)	11 (3.8%)		
Other	24 (8.4%)	18 (6.2%)		
Current AN	5 (1.7%)	6 (2.1%)	0.08	0.01
Current BN	24 (8.4%)	26 (9.0%)	0.07	0.01
Current BED	97 (33.8%)	90 (27.7%)	252	0.06
Current OSFED	10 (3.5%)	18 (6.2%)	2.34	0.06
Current anxiety disorder	88 (30.7%)	104 (36.0%)	1.87	0.05
Current depressive disorder	45 (15.7%)	54 (18.7%)	0.94	0.04
Current substance use disorder	4 (1.4%)	2 (0.7%)	0.68	0.03
Current personality disorder	6 (2.1%)	7 (2.4%)	0.07	0.01
Currently receiving treatment	46 (16.0%)	63 (21.8%)	3.12	0.07
Objective binge eating episodes	14.80 (12.51)	14.98 (10.70)	−0.18	0.01
Eating disorder psychopathology (EDE-Q global)	3.78 (1.00)	3.67 (1.09)	1.28	0.10
Weight concern (EDE-Q subscale)	4.15 (1.17)	4.00 (1.17)	1.56	0.12
Shape concern (EDE-Q subscale)	4.54 (1.18)	4.44 (1.20)	0.98	0.08
Eating concern (EDE-Q subscale)	3.44 (1.24)	3.33 (1.34)	1.00	0.08
Dietary restraint (EDE-Q subscale)	2.99 (1.43)	2.90 (1.57)	0.71	0.06
Subjective binge eating episodes	12.79 (15.48)	13.11 (14.72)	−0.25	0.02
Compensatory behaviors	3.69 (6.56)	3.47 (6.67)	0.40	0.03
Psychological distress (PHQ-4 total)	5.56 (3.03)	5.78 (3.20)	−0.87	0.07
Mindfulness (FFMQ-15 total)	2.89 (0.51)	2.86 (0.51)	0.61	0.05
Emotion regulation (DERS-16 total)	45.21 (14.53)	47.04 (14.62)	−1.50	0.12
Distress tolerance (DTS-4 total)	12.16 (4.33)	12.46 (3.98)	−0.87	0.07

EDE-Q, Eating Disorder Examination Questionnaire; PHQ, Patient Health Questionnaire; FFMQ, Five Facet Mindfulness Scale; DERS, Difficulties in Emotion Regulation Scale; DTS, Distress Tolerance Scale.

Test statistic refers to χ^2^ tests for categorical variables and *t* test for continuous variables; effect size represents Cramér's *V* for categorical variables and Cohen's *d* for continuous variables.

### Attrition

At 6 weeks, 438 participants (76%) provided data on at least one of the primary outcome variables (149/287 [52%] for intervention group and 237/289 [82%] for waitlist) while 238 (41%) provided primary outcome data at 12 weeks (121/287 [42%] for intervention group and 117/289 [40%] for waitlist). The attrition rate at 6 weeks was significantly higher among those allocated to the intervention relative to the waitlist control group (χ^2^ = 58.98, *p* < 0.001; Cramér's *V* = 0.32), but no group differences were found at 12 weeks (χ^2^ = 0.16, *p* = 0.683; Cramér's *V* = 0.01).

Those who did *v.* did not drop out at the two time-points were compared on baseline variables. At 6 weeks, those who dropped out were less likely to report a major depressive disorder at baseline (χ^2^ = 4.13, *p* = 0.042, Cramér's *V* = 0.08), although the effect size was negligible. At 12 weeks, those who dropped out were older (*t* = −2.60, *p* = 0.009, *d* = 0.22) and reported higher baseline objective binge eating episodes (*t* = 2.09, *p* = 0.037, *d* = 0.18), but again the effect sizes were small.

### Engagement

There was variability with engagement of *Resilience* among those allocated to receive immediate access. Over the 6 weeks, the mean number of diary cards completed was 6.96 (s.d. = 8.74), with a range of 0–40 entries. The mean number of skills completed was 4.34 (s.d. = 6.45), with a range of 0–30.0. The percentage of participants who completed modules 1, 2, 3, 4, and 5 was 76%, 61%, 47%, 37%, and 29%, respectively.

### Efficacy at 6 weeks

#### Primary outcomes

Table 2 presents the results from the ITT analyses comparing the intervention and waitlist control groups on primary and secondary outcomes at 6 weeks. The mean differences in objective binge eating (IRR = 0.69) and global eating disorder psychopathology (*d* = −0.71) were statistically significant, with medium-large effect sizes. In both instances, those allocated to the intervention group experienced greater improvements in primary outcomes than the waitlist.

**Table 2.** Means, standard deviations, and change scores on outcomes

	Condition				Difference in change score		
	Waitlist		Intervention		Intervention – waitlist		
Outcome	*n*	*M* (s.d.)	*n*	*M* (s.d.)	*M* [95% CI]	ES	*p*
Objective binge episodes							
Baseline	289	14.98 (10.70)	287	14.80 (12.52)			
6 weeks	237	18.02 (14.24)	149	12.35 (12.15)	0.69 [0.58–0.82]	0.69	<0.001
Eating disorder psychopathology							
Baseline	289	3.67 (1.10)	287	3.78 (1.00)			
6 weeks	237	3.64 (1.13)	149	3.05 (1.16)	−0.71 [−0.88 to −0.53]	−0.68	<0.001
Weight concern							
Baseline	289	4.00 (1.17)	287	4.15 (1.17)			
6 weeks	237	4.05 (1.19)	149	3.49 (1.34)	−0.73 [−0.93 to −0.54]	−0.62	<0.001
Shape concern							
Baseline	289	4.45 (1.21)	287	4.55 (1.19)			
6 weeks	237	4.48 (1.29)	149	3.81 (1.42)	−0.76 [−0.96 to −0.56]	−0.63	<0.001
Dietary restraint							
Baseline	289	2.90 (1.58)	287	2.99 (1.43)			
6 weeks	237	2.87 (1.63)	149	2.35 (1.49)	−0.61 [−0.87 to −0.34]	−0.40	<0.001
Eating concerns							
Baseline	289	3.34 (1.35)	287	3.44 (1.25)			
6 weeks	237	3.18 (1.36)	149	2.56 (1.38)	−0.74 [−0.98 to −0.50]	−0.57	<0.001
Compensatory behaviors							
Baseline	289	3.47 (6.67)	287	3.69 (6.57)			
6 weeks	237	3.93 (8.58)	149	1.71 (3.35)	0.55 [0.40–0.75]	0.55	<0.001
Subjective binge episodes							
Baseline	289	13.11 (14.73)	287	12.79 (15.49)			
6 weeks	237	15.70 (17.71)	149	10.78 (13.23)	0.77 [0.56–1.05]	0.77	0.098
Psychological distress (PHQ-4)							
Baseline	289	5.79 (3.21)	287	5.56 (3.04)			
6 weeks	236	5.91 (3.21)	148	5.24 (3.09)	−0.38 [−0.90 to 0.14]	−0.12	0.151
Mindfulness (FFMQ-15)							
Baseline	289	2.87 (0.52)	287	2.90 (0.51)			
6 weeks	236	2.84 (0.51)	148	2.97 (0.51)	0.11 [0.02–0.19]	0.21	0.013
Emotion regulation (DERS-16)							
Baseline	289	47.04 (14.63)	287	45.22 (14.53)			
6 weeks	236	47.05 (15.25)	147	40.52 (13.77)	−3.99 [−6.11 to −1.88]	−0.27	<0.001
Distress tolerance (DTS-4)							
Baseline	289	12.46 (3.99)	287	12.16 (4.33)			
6 weeks	236	12.46 (4.06)	147	11.74 (4.61)	−0.12 [−1.01 to 0.78]	−0.03	0.797

PHQ-4, Patient Health Questionnaire; FFMQ, Five Facet Mindfulness Scale; DERS, Difficulties in Emotion Regulation Scale; DTS, Distress Tolerance Scale; ES, effect size; 95% CI, 95% confidence interval.

Incident risk ratio for objective binge episodes, subjective binge episodes, and compensatory behaviors, and Cohen's *d* for all other outcomes. *M* and s.d. values are based on non-imputed data; mean differences and effect sizes are derived from ITT analysis (*n* = 587) using multiple imputation.

#### Secondary outcomes

Statistically significant mean differences were observed for the following secondary outcomes: weight concerns (*d* = −0.62), shape concerns (*d* = −0.63), dietary restraint (*d* = −0.40), eating concerns (*d* = −0.57), compensatory behavior frequency (IRR = 0.55), mindfulness (*d* = 0.21), and emotion regulation (*d* = −0.27). In all cases, the intervention group reported greater improvements than the waitlist. No significant group differences were observed for subjective binge eating, psychological distress, and distress tolerance (Table 2).

#### Sensitivity analyses

Online Supplementary Table S2 presents the sensitivity analyses on primary outcomes using different methods of handling missing data. The three different methods of handling missing data produced the same results to the main analyses, but with smaller effect sizes.

### Efficacy at 12 weeks

Table 3 presents the results from the ITT analyses on the degree of change from 6 to 12 weeks. Those allocated to the intervention group experienced further improvements from 6 to 12 weeks on both primary outcomes and on weight concerns, shape concerns, eating concerns, psychological distress, mindfulness, and emotion regulation. Non-significant within-group effects were observed on other secondary outcomes.

**Table 3.** Change scores from 6 to 12 weeks

	Intervention group		Difference in change score			Waitlist group		Difference in change score		
Outcome	*n*	*M* (s.d.)	*M* [95% CI]	ES	*p*	*n*	*M* (s.d.)	*M* [95% CI]	ES	*p*
Objective binge episodes										
6 weeks	149	12.35 (12.15)				237	18.02 (14.24)			
12 weeks	121	10.85 (13.69)	0.80 [0.69–0.92]	0.80	0.002	117	14.80 (16.87)	0.71 [0.61–0.83]	0.71	<0.001
Eating disorder psychopathology										
6 weeks	149	3.05 (1.16)				237	3.64 (1.13)			
12 weeks	121	2.82 (1.18)	−0.32 [−0.47 to −0.17]	−0.28	<0.001	117	2.95 (1.21)	−0.62 [−0.81 to −0.44]	−0.55	<0.001
Weight concern										
6 weeks	149	3.49 (1.34)				237	4.05 (1.19)			
12 weeks	121	3.32 (1.36)	−0.27 [−0.43 to −0.10]	−0.20	0.002	117	3.43 (1.35)	−0.53 [−0.70 to −0.35]	−0.45	<0.001
Shape concern										
6 weeks	149	3.81 (1.42)				237	4.48 (1.29)			
12 weeks	121	3.53 (1.39)	−0.39 [−0.57 to −0.22]	−0.27	<0.001	117	3.67 (1.52)	−0.72 [−0.94 to −0.49]	−0.56	<0.001
Dietary restraint										
6 weeks	149	2.35 (1.49)				237	2.87 (1.63)			
12 weeks	121	2.27 (1.49)	−0.13 [−0.37 to −0.11]	−0.09	0.28	117	2.27 (1.57)	−0.58 [−0.82 to −0.35]	−0.36	<0.001
Eating concerns										
6 weeks	149	2.56 (1.38)				237	3.18 (1.36)			
12 weeks	121	2.15 (1.38)	−0.46 [−0.66 to −0.26]	−0.33	<0.001	117	2.42 (1.29)	−0.68 [−0.90 to −0.46]	−0.50	<0.001
Compensatory behaviors										
6 weeks	149	1.71 (3.35)				237	3.93 (8.58)			
12 weeks	121	1.55 (3.35)	0.77 [0.42–1.42]	0.77	0.402	117	1.65 (4.62)	0.42 [0.26–0.66]	0.42	<0.001
Subjective binge episodes										
6 weeks	149	10.78 (13.23)				237	15.70 (17.71)			
12 weeks	121	9.80 (13.64)	0.96 [0.75–1.23]	0.96	0.747	117	10.56 (14.48)	0.68 [0.53–0.88]	0.68	0.003
Psychological distress (PHQ-4)										
6 weeks	148	5.24 (3.09)				236	5.91 (3.21)			
12 weeks	117	4.79 (3.10)	−0.45 [−0.87 to −0.03]	−0.15	0.034	115	4.91 (3.02)	−0.74 [−1.28 to −0.19]	−0.23	0.008
Mindfulness (FFMQ-15)										
6 weeks	148	2.97 (0.51)				236	2.84 (0.51)			
12 weeks	117	3.08 (0.51)	0.12 [0.05–0.18]	0.24	0.001	115	3.01 (0.49)	0.16 [0.09–0.24]	0.31	<0.001
Emotion regulation (DERS-16)										
6 weeks	147	40.52 (13.77)				236	47.05 (15.25)			
12 weeks	114	39.31 (14.18)	−1.85 [−3.49 to −0.21]	−0.13	0.027	114	41.58 (13.73)	−3.98 [−6.16 to −1.80]	−0.26	<0.001
Distress tolerance (DTS-4)										
6 weeks	147	11.74 (4.61)				236	12.46 (4.06)			
12 weeks	114	11.63 (4.54)	−0.17 [−0.98 to 0.64]	−0.04	0.684	114	11.82 (3.99)	−0.66 [−1.48 to 0.15]	−0.16	0.11

PHQ-4, Patient Health Questionnaire; FFMQ, Five Facet Mindfulness Scale; DERS, Difficulties in Emotion Regulation Scale; DTS, Distress Tolerance Scale; ES, effect size; 95% CI, 95% confidence interval.

Incident risk ratio for objective binge episodes, subjective binge episodes, and compensatory behaviors, and Cohen's *d* for all other outcomes. *M* and s.d. values are based on non-imputed data; mean differences and effect sizes are derived from ITT analysis (*n* = 587) using multiple imputation.

#### Control group

Table 3 also presents the results on the degree of change from 6 to 12 weeks for the waitlist control group who received access to the app during this period. Significant improvements were observed on primary outcomes and on all secondary outcomes except for distress tolerance.

### Negative effects

There were 259 participants who responded to negative effects items. Eleven (4.2%) rated ‘yes’ to the item asking whether the app led to an aggravation of previously experienced symptoms; responses largely indicated heighted symptoms related to greater awareness or preoccupation with eating, more intense urges, and feeling de-motivated after not seeing progress. Four (1.5%) responded ‘yes’ to the item asking about the emergence of new symptoms, with responses indicating feelings of hopelessness and abandonment.

## Discussion

We conducted the first RCT evaluating the efficacy of a DBT skills training app for individuals with recurrent binge eating. We found that participants allocated to the intervention experienced significantly greater improvements in primary outcomes, most secondary symptom measures, and two of the three purported DBT mechanisms relative to the waitlist control group at 6 weeks. Critically, primary symptoms continued to improve from 6 to 12 weeks. There was minimal evidence that the app incurred harm, which is an important finding that stands in contrast to recent trials identifying iatrogenic effects of online DBT programs offered to other clinical populations (Simon et al., 2022). Findings highlight the clinical efficacy of a novel app-based DBT intervention designed for the management of eating disorder symptoms.

The current findings add to growing literature highlighting the clinical utility of app interventions for eating disorders. The magnitude of effects on primary outcomes closely aligns with effect sizes observed in recent trials that delivered CBT self-management apps to individuals with recurrent binge eating (Linardon et al., 2023). This finding reinforces prior claims that DBT could be considered an empirically supported alternative to current front-line treatments (Vogel, Singh, & Accurso, 2021). Understanding when, for whom, and under what circumstances DBT approaches may be the most suitable option is a crucial next step. It could be that DBT is most useful among those who (i) fail to achieve a rapid early response to CBT, (ii) exhibit signs of premature treatment discontinuation, or (iii) express strong preference for approaches that emphasize emotion regulation rather than direct behavior change. Capitalizing on adaptive clinical trial designs (Ryan et al., 2024) that involve repeated re-randomization to different treatment options based on ongoing user progress, needs, and feedback may prove useful for understanding the optimal conditions under which DBT approaches are most suited.

The app also produced broader effects beyond reductions in binge eating, with improvements found on body image concerns, compensatory behaviors, and dietary restraint. Given that these symptoms are also precipitated by adverse emotional states (Lavender et al., 2015), it is possible that participants were successfully able to generalize their use of DBT skills to other contexts for which these symptoms typically emerge (e.g. engaging in mindful awareness during periods of self-critical thoughts pertaining to body shape). Alternatively, perhaps improvements in binge eating have a cascade effect on those other symptoms that are thought to maintain it. This interpretation is consistent with those theoretical models that emphasize the self-perpetuating nature of eating disorder psychopathology (Fairburn, Cooper, & Shafran, 2003), where it is assumed that successfully targeting one symptom would indirectly affect the others implicated in the cycle.

The app also had a positive effect on two of the three purported change mechanisms of DBT interventions. We observed small, significant effects of increased mindfulness and emotion regulation skills (but not distress tolerance) in favor of the intervention over the waitlist control group. This finding is important because it provides the necessary foundational evidence required to identify the working mechanisms of an intervention (Lorenzo-Luaces, German, & DeRubeis, 2014), offering a rationale for future research to employ intensive longitudinal designs to test whether this app exerts its effects by modifying these constructs. Future research would benefit from (i) measuring these putative mechanisms and symptoms weekly during the course of the intervention so that their trajectory of change can be mapped and analyses that permit inferences of temporal precedence can performed, or (ii) manipulating these mechanisms and assessing their casual impacts through rigorous trial designs, such as the dismantling or factorial trial.

Problems with attrition and engagement were observed. Less than 50% of participants allocated to the intervention group completed the 6 and 12 week assessments, while only one-third accessed the final module. These problems are widespread in digital health trials (Linardon & Fuller-Tyszkiewicz, 2020) and there may be a number of reasons for this. First, this trial was automated and fully remote, so it was not possible to develop rapport with participants, set up a commitment to engage, or ensure that each person fully understood the requirements for participation. Second, professional guidance was not offered, so participants may have lacked a sense of accountability or motivation when engaging with the therapeutic content and mastering it. Third, no reimbursement was provided, which has been shown to increase rates of retention and engagement by nearly 20% (Linardon & Fuller-Tyszkiewicz, 2020). Future trials may circumvent these problems by offering in-person or telephone screening/assessments, providing an app that offers either professional or automated personalized support, or incentivizing participants.

Limitations to this study must be considered. First, the use of a passive control group is a limitation because it has the potential to inflate efficacy estimates via the digital placebo effect (Torous & Firth, 2016). Some contend that waitlists may be better conceptualized as a nocebo condition because the uncertainty of waiting for intervention may exacerbate symptoms and lead to negative expectations about recovery (Furukawa et al., 2014). Indeed, waitlist participants' objective binge eating frequency, for example, increased from a mean of 14 episodes at baseline to 18 episodes at 6 weeks, suggesting that the combination of improvement in intervention participants and deterioration in waitlist participants may have inflated our effect size estimates on certain outcomes. A future direction would be to compare *Resilience* to a credible comparison condition. We have generated evidence for the efficacy of the *Break Binge Eating* CBT app (not publicly available) in the same target population (Linardon et al., 2023), so pitting *Resilience* against *Break Binge Eating* could help to not only establish their relative efficacy, but to also identify critical moderators of response necessary for realizing the potential of personalized medicine. Second, as with all fully remote digital health trials (Linardon & Fuller-Tyszkiewicz, 2020), we had difficulty retaining a significant participants despite the provision of evidence-informed retention strategies (e.g. gamification, notifications). However, results remained unchanged in sensitivity analyses that handled missing data in various ways. Third, given that the sample was mostly White, findings cannot be generalized to people of different racial or ethnic backgrounds. It is important for future research testing novel digital health tools to diversify their sample so that we can better understand for whom may be most or least suited to these intervention formats.

The present study adds to an emerging body of evidence demonstrating the clinical benefit of smartphones apps for the management of eating disorders. We found that a low intensity, stand-alone, DBT-based app led to improvements in core symptoms of eating disorders. The present findings suggest that such an app could be an appropriate intervention option for those who cannot access traditional forms of treatment. Alternatively, an app like this could potentially be delivered to those placed on a waiting list to assist with short-term symptom relief or to help the person build foundational knowledge, skills, and motivation required to support the recovery process. As we have intentions to make *Resilience* publicly available in the app store, evaluating the many roles an app like this may play in the clinical care for eating disorders will be a priority in coming years.

## Supporting information

Linardon et al. supplementary material Linardon et al. supplementary material

## Supplementary material

The supplementary material for this article can be found at https://doi.org/10.1017/S0033291724002800.
