# Supplementary material for: A dialectical behavior therapy skills training smartphone app for recurrent binge eating: a randomized clinical trial
Source: Psychol Med. 2024 Dec 9;54(16):4646–57. doi: 10.1017/S0033291724002800 (PMC11773374; doi:10.1017/S0033291724002800)
Supplement: Linardon et al. supplementary material [file S0033291724002800sup001.docx]

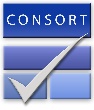


CONSORT 2010 checklist of information to include when reporting a randomised trial*

| Section/Topic | Item No | Checklist item | Reported on page No |
| --- | --- | --- | --- |
| Title and abstract | | | |
|  | 1a | Identification as a randomised trial in the title | 1 |
|  | 1b | Structured summary of trial design, methods, results, and conclusions (for specific guidance see CONSORT for abstracts) | 2 |
| Introduction | | | |
| Background and objectives | 2a | Scientific background and explanation of rationale | 3-5 |
|  | 2b | Specific objectives or hypotheses | 5 |
| Methods | | | |
| Trial design | 3a | Description of trial design (such as parallel, factorial) including allocation ratio | 6 |
|  | 3b | Important changes to methods after trial commencement (such as eligibility criteria), with reasons | 6 |
| Participants | 4a | Eligibility criteria for participants | 6 |
|  | 4b | Settings and locations where the data were collected | 6 |
| Interventions | 5 | The interventions for each group with sufficient details to allow replication, including how and when they were actually administered | 7 |
| Outcomes | 6a | Completely defined pre-specified primary and secondary outcome measures, including how and when they were assessed | 9 |
|  | 6b | Any changes to trial outcomes after the trial commenced, with reasons | 6 |
| Sample size | 7a | How sample size was determined | 10-11 |
|  | 7b | When applicable, explanation of any interim analyses and stopping guidelines | Not applicable |
| Randomisation: |  |  |  |
| Sequence generation | 8a | Method used to generate the random allocation sequence | 7 |
|  | 8b | Type of randomisation; details of any restriction (such as blocking and block size) | 7 |
| Allocation concealment mechanism | 9 | Mechanism used to implement the random allocation sequence (such as sequentially numbered containers), describing any steps taken to conceal the sequence until interventions were assigned | 7 |
| Implementation | 10 | Who generated the random allocation sequence, who enrolled participants, and who assigned participants to interventions | 7 |
| Blinding | 11a | If done, who was blinded after assignment to interventions (for example, participants, care providers, those assessing outcomes) and how | 7 |
|  | 11b | If relevant, description of the similarity of interventions | 7-9 |
| Statistical methods | 12a | Statistical methods used to compare groups for primary and secondary outcomes | 11-12 |
|  | 12b | Methods for additional analyses, such as subgroup analyses and adjusted analyses | 11-13 |
| Results | | | |
| Participant flow (a diagram is strongly recommended) | 13a | For each group, the numbers of participants who were randomly assigned, received intended treatment, and were analysed for the primary outcome | Figure 1 |
|  | 13b | For each group, losses and exclusions after randomisation, together with reasons | Figure 1 |
| Recruitment | 14a | Dates defining the periods of recruitment and follow-up | 6 |
|  | 14b | Why the trial ended or was stopped | 6 |
| Baseline data | 15 | A table showing baseline demographic and clinical characteristics for each group | Table 1 |
| Numbers analysed | 16 | For each group, number of participants (denominator) included in each analysis and whether the analysis was by original assigned groups | Tables 2-3 |
| Outcomes and estimation | 17a | For each primary and secondary outcome, results for each group, and the estimated effect size and its precision (such as 95% confidence interval) | Tables 2-3 |
|  | 17b | For binary outcomes, presentation of both absolute and relative effect sizes is recommended | Not applicable |
| Ancillary analyses | 18 | Results of any other analyses performed, including subgroup analyses and adjusted analyses, distinguishing pre-specified from exploratory | 14 |
| Harms | 19 | All important harms or unintended effects in each group (for specific guidance see CONSORT for harms) | 14-15 |
| Discussion | | | |
| Limitations | 20 | Trial limitations, addressing sources of potential bias, imprecision, and, if relevant, multiplicity of analyses | 17-18 |
| Generalisability | 21 | Generalisability (external validity, applicability) of the trial findings | 18 |
| Interpretation | 22 | Interpretation consistent with results, balancing benefits and harms, and considering other relevant evidence | 15-16 |
| Other information | | |  |
| Registration | 23 | Registration number and name of trial registry | 6 |
| Protocol | 24 | Where the full trial protocol can be accessed, if available | 6 |
| Funding | 25 | Sources of funding and other support (such as supply of drugs), role of funders | 1 |

| **Table S2**  **Sensitivity Analyses on Primary Outcomes** | | | | | | | | | |  |
| --- | --- | --- | --- | --- | --- | --- | --- | --- | --- | --- |
|  |  | **Control** | | | **Intervention** | | | **Difference in change score** | | |
| **Method** | **Primary Outcome** | ***n*** | ***M*** | ***SD*** | ***n*** | ***M*** | ***SD*** | **M (95% CI)** | **ES** | ***p*** |
| LMFC |  |  |  |  |  |  |  |  |  |  |
|  | Objective binge eating |  |  |  |  |  |  |  |  |  |
|  | Baseline | 289 | 14.98 | 10.70 | 287 | 14.80 | 12.52 |  |  |  |
|  | Six weeks | 237 | 18.02 | 14.24 | 149 | 12.35 | 12.15 | 0.78 (0.67, 0.91) | 0.78 | .001 |
|  | EDE-Q global |  |  |  |  |  |  |  |  |  |
|  | Baseline | 289 | 3.67 | 1.10 | 287 | 3.78 | 1.00 |  |  |  |
|  | Six weeks | 237 | 3.64 | 1.13 | 149 | 3.05 | 1.16 | -0.36 (-0.52, -0.20) | -0.34 | <.001 |
| J2R |  |  |  |  |  |  |  |  |  |  |
|  | Objective binge eating |  |  |  |  |  |  |  |  |  |
|  | Baseline |  |  |  |  |  |  |  |  |  |
|  | Six weeks |  |  |  |  |  |  | 0.82 (0.70, 0.97) | 0.82 | .017 |
|  | EDE-Q global |  |  |  |  |  |  |  |  |  |
|  | Baseline |  |  |  |  |  |  |  |  |  |
|  | Six weeks |  |  |  |  |  |  | -0.43 (-0.59, -0.27) | -0.41 | <.001 |
| CIR |  |  |  |  |  |  |  |  |  |  |
|  | Objective binge eating |  |  |  |  |  |  |  |  |  |
|  | Baseline |  |  |  |  |  |  |  |  |  |
|  | Six weeks |  |  |  |  |  |  | 0.82 (0.70, 0.97) | 0.82 | .021 |
|  | EDE-Q global |  |  |  |  |  |  |  |  |  |
|  | Baseline |  |  |  |  |  |  |  |  |  |
|  | Six weeks |  |  |  |  |  |  | -0.37 (-0.52, -0.21) | -0.35 | <.001 |
| LMFC = Last mean carried forward; J2R = Jump to reference; CIR = copy increments in reference; Effect size; Relative Risk for objective binge episodes and Cohen’s *d* for EDE-Q global scores. | | | | | | | | | | |
